# Electrocardiographic reference values and configuration of electrocardiogram waves recorded in Black Bengal goats of different age groups

**DOI:** 10.14202/vetworld.2017.1020-1025

**Published:** 2017-09-02

**Authors:** Ranjeeta Rashmee Pradhan, Ambika Prasad Khadanga Mahapatra, Swagat Mohapatra, Tushar Jyotiranjan, Akshaya Kumar Kundu

**Affiliations:** Department of Veterinary Physiology, College of Veterinary Science and Animal Husbandry, Orissa University of Agriculture and Technology, Bhubaneswar - 751 003, Odisha, India

**Keywords:** age, Black Bengal goats, electrocardiogram

## Abstract

**Aim:**

A study on age-related electrocardiographic (ECG) changes was conducted on 20 apparently healthy Black Bengal goats with no history of cardiac disorders during 2015-2016.

**Materials and Methods:**

The goats selected for the study belonged to four different age groups; Group 1: Goats up to 6 months of age, Group 2: Above 6 months and below 1 year of age, Group 3: Above 1 year and below 2 years of age, and Group 4: Above 2 years of age. The ECG was recorded with the animals in standing position using a 12-lead standard ECG recorder (Model-Cardiart-108 MK VII, manufactured by BPL, India). The paper speed was set to 25 mm/s with the sensitivity of the machine was adjusted at 1 (1 cm=mV).

**Results:**

The ECG parameters were compared within different age groups, and the data were analyzed statistically using SPSS 16.0 taking a significant level of 95% (p<0.05) in all cases. The lead-I ECG revealed a significant difference in amplitude of QRS complex, PR interval, QT interval, RR interval, PQ segment, ST segment, TP segment, and heart rate among some age groups. In bipolar limb lead-II, the amplitude of T-wave, RR interval, ST segment, TP segment, and heart rate was a significant difference among some age groups. Lead-III presented significant difference among age groups in different parameters such as QRS complex duration, T-wave duration, RR interval, ST segment, TP segment, and heart rate.

**Conclusion:**

The study concluded that there is a significant variation in the ECG parameters both in terms of values and configuration of ECG waves when age is taken into consideration. The results of the study might be used as a reference value for field veterinarians.

## Introduction

An electrocardiogram (ECG) is the recording of electric potentials generated by the cardiac impulse by placing electrodes on the skin on opposite sides of the heart [1]. Research works on caprine ECG are rare in comparison to numerous data available for normal physiological cardiac arrhythmias in bovine [2] and ovine species [3]. In spite of many scientific advances, the goal of reducing cardiovascular morbidity and mortality remains as one of the most vexing challenges.

Considering the perspective of clinical treatment, a correct ECG diagnosis can only be done if the ECG values of the screened goats are compared with normal reference values established in healthy goats. The size and shape of the hearts in goats vary according to its breed and body size, and this variation is expected to be reflected in the ECG [4]. The Black Bengal goat is a breed of goat found throughout Bangladesh and other places of India such as West Bengal, Bihar, and Odisha. One of the most favorable attributes of the Black Bengal goat as a meat-producing animal is its high rate of reproduction and the fact that it has an extended breeding, especially as reproduction is a major contributing factor to the efficiency of meat production [5]. This breed can adapt to any environment easily and its immunity is very high.

As per our knowledge, there is no report on ECG change with advancing age in the Black Bengal goat. Therefore, the present study was conducted to assess the alterations in ECG parameters of Black Bengal goats in different age groups which may act as a source of reference for veterinary clinicians while interpreting the ECG.

## Materials and Methods

### Ethical approval

Approval of Animal Ethics Committee is not required in no-invasive types of experiments.

### Experimental design

The animals were assigned into four groups according to their age, comprising Group 1 (up to 6 months of age, n=6), Group 2 (above 6 months and below 1 year of age, n=6), Group 3 (above 1 year and below 2 years of age, n=6), and Group 4 (above 2 years of age, n=6). The animals were examined before ECG recordings. The animals were examined for clinical signs such as coughing, paleness of mucous membrane, fatigue, shortness of breath, and swollen abdomen. The animals showing no such clinical signs and history of cardiac disorders were selected for the study. A 12-lead standard ECG recorder, Cardiart 108 T-MK VII- BPL, India, was used to record ECG in all the goats. ECG was taken with the animal restrained in standing position on a wooden board. The ECG machine was set for a paper speed of 25 mm/s and sensitivity of 1 (1 cm=1 mv) with the 50 cycles filter turned “on.” The anterolateral aspect, just below the elbow, and stifle joint were the preferred sites of attachment of crocodile clips, and the ECG tracings were taken in three bipolar standard leads (leads I, II, and III) as described by Ahmed and Sanyal [6]. The ECG values of different age groups were compared taking all the bipolar limb leads into consideration.

### Statistical analysis

The parameters were compared using MS Excel and SPSS-10 software.

## Results

**Figure-1:**
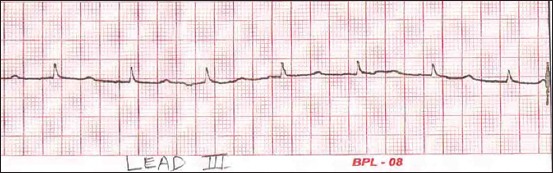
Sample lead-III electrocardiogram of Black Bengal goat (<6 months) of age (Group A).

The results mentioned here are in accordance to Tables-1-3. Analysis of variance revealed that none of the bipolar limb leads recorded a significant difference between the P-wave amplitudes of different age groups. The measured values of P-wave amplitude were invariably the same in all the leads. The configuration of the P-waves in different leads and age groups is mentioned in Table-4. The configuration of P-wave in Group A recorded positively (above the isoelectric baseline) in all the leads. Similarly, amplitude of P-wave in all the groups recorded positively in lead-III (Figure-1), whereas inverted P-waves were observed in lead-I and lead-II of Groups B and C. Upright P-waves were observed in lead-I and lead-III of Group D, whereas lead-II recorded inverted P-waves in all animals of Group D. The duration of P-wave had no significant difference among the groups in leads I and III. While in lead-II, the P-wave duration was found to be significantly higher in Group D.

**Table-1:** Cardiovascular changes in lead-I of Black Bengal goats in different age groups.

Age group	P-wave amplitude (mV)	P-wave duration (s)	QRS amplitude (mV)	QRS duration (s)	T-wave amplitude (Mv)	T-wave duration (s)	P-R interval (s)	Q-T interval (s)	R-R interval (s)	P-Q segment (s)	S-T segment (s)	T-P segment (s)	Heart rate (bpm)
Group A	0.1±0^a^	0.04±0^a^	0.52±0.07^a^	0.056±0.138^a^	0.1±0^a^	0.06±0.01^a^	0.04±0^a^	0.27±0.01^a^	0.41±0.01^a^	0.05±0.01^a^	0.23±0.005^a,b^	0.22±0.01^a^	147.26±2.74^a^
Group B	0.1±0^a^	0.04±0^a^	0.56±0.04^a^	0.056±0.138^a^	0.2±0.03^a^	0.09±0.01^a^	0.10±0.01^b^	0.34±0.01^b^	0.46±0.01^b^	0.06±0.01^a^	0.24±0.01^a,b^	0.39±0.01^b^	129.52±2.77^b^
Group C	0.1±0^a^	0.04±0^a^	0.15±0.01^b^	0.056±0.138^a^	0.18±0.04^a^	0.06±0.02^a^	0.09±0.01^b^	0.30±0.01^a^	0.46±0.02^b^	0.07±0.01^a^,^b^	0.18±0.06^a^	0.35±0.01^c^	129.78±4.29^b^
Group D	0.1±0^a^	0.04±0^a^	0.48±0.04^a^	0.056±0.138^a^	0.16±0.02^a^	0.08±0.01^a^	0.10±0.01^b^	0.34±0.01^b^	0.49±0.01^b^	0.10±0.01^b^	0.29±0.01^b^	0.27±0.01^d^	123.0±2^b^

Group A: Goats up to 6 months, Group B: Goats above 6 months and below 1 year of age, Group C: Goats above 1 year and below 2 years of age, Group D: Goats above 2 years of age. Values bearing different superscripts in a column differ significantly (p<0.05)

**Table-2:** Cardiovascular changes in lead-II of Black Bengal goats in different age groups.

Age group	P-wave amplitude (mV)	P-wave duration (s)	QRS amplitude (mV)	QRS duration (s)	T-wave amplitude (Mv)	T-wave duration (s)	P-R interval (s)	Q-T interval (s)	R-R interval (s)	P-Q segment (s)	S-T segment (s)	T-P segment (s)	Heart rate (bpm)
Group A	0.1±0^a^	0.04±0^a^	0.20±0.04^a^	0.06±0.01^a^	0.1±0.0^a^	0.05±0.01^a^	0.10±0.01^a^	0.24±0.01^a^	0.37±0.01^a^	0.05±0.01^a^	0.26±0.01^a^	0.29±0.01^a^	163.6±3.4^a^
Group B	0.1±0^a^	0.04±0^a^	0.24±0.04^a^	0.07±0.01^a^	0.18±0.04^a,b^	0.06±0.01^a^	0.09±0.01^a^	0.26±0.01^a^	0.52±0.01^b^	0.05±0.01^a^	0.26±0.01^a^	0.38±0.03^b^	115.4±2.86^b^
Group C	0.1±0^a^	0.04±0^a^	0.20±0.01^a^	0.06±0.01^a^	0.18±0.040^a,b^	0.06±0.02^a^	0.09±0.01^a^	0.22±0.02^a^	0.54±0.01^b^	0.06±0.01^a,b^	0.25±0.01^a^	0.50±0.01^c^	110.2±1.96^b^
Group D	0.1±0^a^	0.07±0.01^b^	0.22±0.03^a^	0.06±0.01^a^	0.22±0.040^b^	0.09±0.04^b^	0.11±0.01^a^	0.25±0.01^a^	0.54±0.02^b^	0.08±0^b^	0.30±0.01^b^	0.51±0.01^c^	112.2±3.56^b^

Group A: Goats up to 6 months, Group B: Goats above 6 months and below 1 year of age, Group C: Goats above 1 year and below 2 years of age, Group D: Goats above 2 years of age. Values bearing different superscripts in a column differ significantly (p<0.05)

**Table-3:** Cardiovascular changes in lead-III of Black Bengal goats in different age groups.

Age group	P-wave amplitude (mV)	P-wave duration (s)	QRS amplitude (mV)	QRS duration (s)	T-wave amplitude (Mv)	T-wave duration (s)	P-R interval (s)	Q-T interval (s)	R-R interval (s)	P-Q segment (s)	S-T segment (s)	T-P segment (s)	Heart rate (bpm)
Group A	0.1±0^a^	0.04±0^a^	0.39±0.05^a^	0.05±0.01^a^	0.1±0^a^	0.04±0^a^	0.08±0.02^a^	0.22±0.01^a^	0.38±0.01^a^	0.08±0^a^	0.30±0.01^a,b^	0.30±0.03^a,b^	156.8±4.16^a^
Group B	0.1±0^a^	0.04±0^a^	0.53±0.03^a^	0.05±0.01^a^	0.1±0^a^	0.07±0.01^b^	0.12±0^a^	0.26±0.01^a^	0.42±0.01^a,b^	0.08±0^a^	0.31±0.01^b^	0.28±0^a^	141.6±3.43^b^
Group C	0.1±0^a^	0.04±0^a^	0.55±0.09^a^	0.06±0.01^a,b^	0.1±0^a^	0.07±0.01^b^	0.10±0.02^a^	0.28±0^a^	0.50±0.02^b,c^	0.08±0^a^	0.31±0.01^b^	0.36±0^b^	117.4±3.43^c^
Group D	0.1±0^a^	0.04±0^a^	0.40±0.04^a^	0.08±0.01^b^	0.1±0^a^	0.07±0.01^b^	0.09±0.01^a^	0.28±0^a^	0.47±0.02^c^	0.08±0^a^	0.28±0^a^	0.30±0.02^a,b^	119±2.45^c^

Group A: Goats up to 6 months, Group B: Goats above 6 months and below 1 year of age, Group C: Goats above 1 year and below 2 years of age, Group D: Goats above 2 years of age. Values bearing different superscripts in a column differ significantly (p<0.05)

**Table-4:** Configuration of P-wave in Black Bengal goats.

Configuration of P-wave	Up to 6-month-old Black Bengal goats (Group A)			Above 6-month and below 1-year-old Black Bengal goats (Group B)			Above 1 and below 2-year-old Black Bengal goats (Group C)			Above 2-year-old Black Bengal goats (Group D)		
			
	Lead I	Lead II	Lead III	Lead I	Lead II	Lead III	Lead I	Lead II	Lead III	Lead I	Lead II	Lead III
Inverted				6	6		6	6			6	
Upright	6	6	6			6			6	6		6
Total	6			6			6			6		

In this study, the amplitude of QRS complex had no significant difference among the different age groups in lead-II and lead-III, whereas in lead-I, the QRS complex amplitude of Group C animals was significantly lower than the other groups. The configuration of QRS complex varied widely as indicated in Table-5. In limb lead-I, the duration of QRS complex did not vary significantly within groups. However, in lead-II, the QRS duration was found to be non-significantly higher in Group B. Similarly, in lead-III, the QRS duration of Group D was found to be highest.

**Table-5:** Configuration of QRS complex in Black Bengal goats.

Configuration of QRS complex	Up to 6-month-old Black Bengal goats (Group A)			Above 6-month and below 1-year-old Black Bengal goats (Group B)			Above 1 and below 2-year-old Black Bengal goats (Group C)			Above 2-year-old Black Bengal goats (Group D)		

	Lead I	Lead II	Lead III	Lead I	Lead II	Lead III	Lead I	Lead II	Lead III	Lead I	Lead II	Lead III
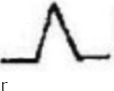	4	2			1	1	2	3		1	1	1
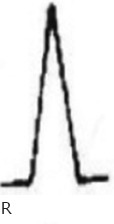	2	3	3	6	3	5	3		3	4	4
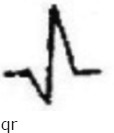		1	2		1			3			4	1
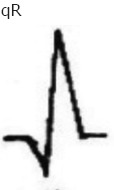									3			
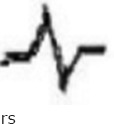										1		
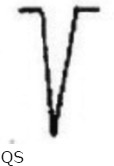			1		1		1				1	

**Figure-2:**
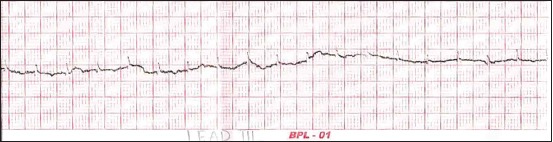
Sample lead-III electrocardiogram of Black Bengal goat (>6 months <1 year) of age (Group B).

The T-wave amplitude of Black Bengal goats belonging to different age groups did not reveal any significant difference between age groups in lead-I and lead-III. However, in lead-II, the amplitude of T-wave varied significantly within the group, and the highest amplitude was measured in Group D. The duration of T-wave belonging to different age groups was found to be statistically insignificant in lead-I. However, in lead-II, the duration of T-wave was significantly higher in Group D, and in lead-III, significantly higher amplitudes were obtained in Groups B (Figure-2), C, and D in comparison to Group A.

There was no significant difference between PR interval and QT interval in lead-II and lead-III. However, in lead-I, both the PR interval and QT interval were found to be significantly lower in Group A. R-R interval in Black Bengal goat shows no significant difference between lead-I and lead-II, whereas in lead-III, the R-R interval has a significant difference within the group.

**Figure-3:**
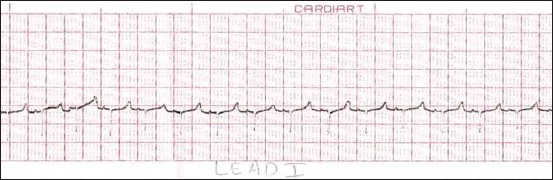
Sample lead-I electrocardiogram of Black Bengal goat (>1 year <2 years) of age (Group C).

**Figure-4:**
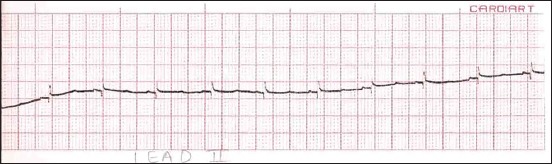
Sample lead-II electrocardiogram of Black Bengal goat (>2 years) of age (Group D).

Group D recorded significantly higher PQ segment in lead-I and lead-II, whereas in lead-III, there was no significant difference within groups. In Black Bengal goat, the ST segment was recorded as significantly different in lead-I, lead-II, and lead-III. There was a significant difference between groups with regard to TP segment of Black Bengal goat in all the leads. Lead-I and lead- II electrocardiogram tracings of Group C and Group D are shown in Figures 3 and 4 respectively.

Significantly higher heart rates were invariably observed in the youngest goats, i.e., Group A in all the leads taken into consideration.

## Discussion

Our observations are not in agreement with Atmaca *et al*. [7], who recorded positive P-waves in all leads except aVR and aVL in Angora goats aged between 1 and 2 years. The variation in the configuration of P-waves in these two breeds might be attributed to the breed-specific difference in the pattern of atrial depolarization. The duration of P-wave recorded no significant difference within the groups in the bipolar limb leads I and III, but in lead-II, the duration of P-wave was significantly higher in Group D as compared to other groups indicating slight dilatation of atria with increased age.

There was no statistical significance between the amplitudes of QRS complex in leads II and III. However, the QRS complex amplitude was significantly lower (p<0.05) in Group C of lead-I, as compared to other age groups. The low amplitude QRS complex might be due to high degree of synchronized ventricular depolarization [8]. The variation in configuration might be attributed to the fact that the mean electrical axis, i.e., the general direction in which the depolarization waves spread through the ventricles, varied between +115° and +192°. Such wide range of axis orientation was also reported by Ahmed and Sanyal [6] in Black Bengal goats. The cardiac electrical axis undergoes a left rotation in day and month old kids [9]. The change in cardiac axis as age advances is apparently the reason behind different configurations of QRS complex in different age groups. The observed QRS duration of Black Bengal goat in this study is similar to the duration recorded in Markhor goat [10].

The amplitude of T-wave of Black Bengal goats belonging to different age groups did not reveal any significant difference between age groups in lead-I and lead-III. However, in lead-II, the amplitudes of T-wave varied significantly within groups, and the highest amplitude was measured in Group D. T-wave amplitude recorded positively in all leads. The present finding is in agreement with Ahmed and Sanyal [6].

The mean duration of T-wave belonging to different age groups was found to be statistically insignificant in lead-I. However, in lead-II, the duration of T-wave was significantly higher in Group D, and in lead-III, significantly higher values were obtained in Groups B, C, and D in comparison to Group A. Our values are not in agreement with Mohapatra *et al*. [11], who reported no significant difference between adult sheep and lambs.

The PR interval denotes the atrioventricular conduction time. There was no significant difference between PR intervals in lead-II and lead-III, whereas Group A exhibited a significantly lower (p<0.05) PR interval in lead-I. Shorter PR interval results in shorter interval between successive cardiac cycles. Mir *et al*. [12] also reported shorter PR intervals in lambs as compared to adult sheep. In leads II and III, QT interval did not vary significantly within groups of Black Bengal goat. However, in lead-I, QT interval was found to be significantly different among groups. Wide variation in QT interval may be due to its dependence on age, sex, and heart rate [13]. R-R interval was found to be significantly lowest in Group A of all the leads taken into consideration. Variation in the rate and rhythm not only can occur in normal animals due to strong or varying autonomic influence but also can be an indication of myocardial disease. Since there were no other indications of myocardial damage in the ECG, we assume that the variations in rate and rhythm are normal. There are some other factors such as acid-base and electrolyte imbalances that can influence rate and rhythm [14].

The PQ segment was found to be significantly higher in older animals (Groups C and D) as compared to younger ones (Groups A and B) in leads I and II, whereas no significant difference was observed amidst groups in lead-III. Analysis of variance revealed that there was a significant difference of ST segment in leads I, II, and III. In lead-I, it was found that younger goats had higher ST segment than older ones, while ST segment in lead-II and lead-III was higher in older animals. The TP segment of Black Bengal goat in different leads differed significantly within age groups.

Significantly higher heart rates were invariably observed in the youngest animals, i.e., Group A in all the leads taken into consideration. Higher heart rates in kids might be due to stress and excitation caused by separation of kids from their does [15].

## Conclusion

The study concludes that age has direct influence on the outcome of values of ECG parameters as well as, the configuration of different ECG waves varies with respect to age and also within the same age group of goats. So, while analyzing ECG in apparently healthy Black Bengal goats, all these factors should be taken into consideration for proper or an accurate interpretation of cardiac originated problems from the ECG only.

## Authors’ Contributions

The work was carried out, and the manuscript was written by RRP as a part of her M.V.Sc. research program, APKM was the chairman of the advisory committee. RRP, APKM, SM, and AKK designed the work. RRP, SM, and TJ performed the field work of recording ECG. RRP and TJ performed the statistical analysis. SM edited the article. AKK and APKM made the final revision of the manuscript. All authors read and accepted the final manuscript.
